# 500. Durability of antibody response after booster vaccination against SARS-CoV-2 in kidney transplant recipients with or without prior infection

**DOI:** 10.1093/ofid/ofad500.569

**Published:** 2023-11-27

**Authors:** Thornthun Ueaphongsukkit, Athiphat Banjongjit, Yingyos Avihingsanon, Suwasin Udomkarnjananun, Natavudh Townamchai, Kamonwan Jutivorakool, Jakapat Vanichanan

**Affiliations:** Division of Infectious Diseases, Department of Medicine, Faculty of Medicine, Chulalongkorn University and King Chulalongkorn Memorial Hospital, Bangkok, Thailand, Pathumwan, Krung Thep, Thailand; Division of Nephrology, Department of Medicine, Faculty of Medicine, Chulalongkorn University, Bangkok, Thailand, Pathumwan, Krung Thep, Thailand; Division of Nephrology and Renal Immunology and Renal Transplant Research Unit, Department of Medicine, Faculty of Medicine, Chulalongkorn University and Excellence Center for Organ Transplantation, King Chulalongkorn Memorial Hospital, Bangkok, Thailand., Pathumwan, Krung Thep, Thailand; King Chulalongkorn Memorial Hospital, Bangkok, Krung Thep, Thailand; Division of Nephrology and Renal Immunology and Renal Transplant Research Unit, Department of Medicine, Faculty of Medicine, Chulalongkorn University and Excellence Center for Organ Transplantation, King Chulalongkorn Memorial Hospital, Bangkok, Thailand., Pathumwan, Krung Thep, Thailand; King Chulalonkorn Memorial Hospital, Bangkok, Krung Thep, Thailand; Chulalongkorn University, Bangkok, Krung Thep, Thailand

## Abstract

**Background:**

Kidney transplant recipients (KTR) are at increased risk of severe COVID-19 infection due to their immunosuppressive drugs. COVID-19 vaccination is recommended to prevent infection and severe illness. However, the knowledge on the durability of vaccine response and appropriate timing of booster vaccination is not well established. This study aimed to evaluate the anti-SARS-CoV-2 S antibody response > 12 weeks after the fourth vaccination and to identify factors associated with a good antibody response in KTR.

**Methods:**

This single-center cross-sectional study was conducted at King Chulalongkorn Memorial hospital in Bangkok, Thailand between January and April 2023. It enrolled adult KTR who had been transplanted for more than 6 months and had received the fourth COVID-19 vaccine dose for more than 12 weeks. KTR with prior long acting antibody (LAAB) were excluded. Baseline characteristics, laboratory data, COVID-19 vaccine certificates, and history of prior infection were collected. Blood samples were taken at enrollment for anti-SARS-CoV-2 S antibody testing (Elecsys®, Cobas e 411 analyzer; Roche Diagnostics, Basel, Switzerland). An antibody level ≥ 1,000 BAU/mL was considered a high sustained response. All statistical analyses were performed using Stata 15.1 (Stata Corp., College Station, TX, USA).

**Results:**

A total of 132 KTR were enrolled. The median age was 52 years, and 60% were male. Most KTR received two doses of viral vector vaccine as their first two primary shots with two doses of mRNA vaccine as the additional primary and booster shots. The overall anti-SARS-CoV-2 S antibody level was 4,917 (IQR 884.4 – 16,888.5) BAU/mL at the median of 261.5 (IQR 202 – 297.5) days since last vaccination. There were 96 (73%) of KTR with antibody level ≥ 1,000 BAU/mL. Baseline characteristics were shown in Table 1. History of COVID-19 infection was significantly associated with a good antibody response after multivariate logistic regression analysis with adjusted OR of 3.87 (95% CI 1.64 – 9.08) (Figure 1).

**Figure:**
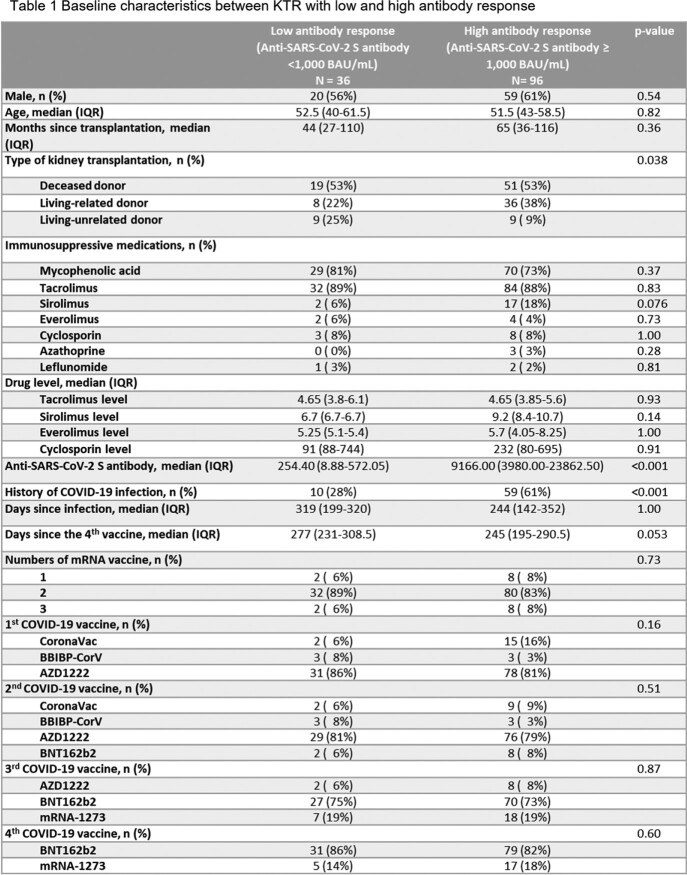

**Figure:**
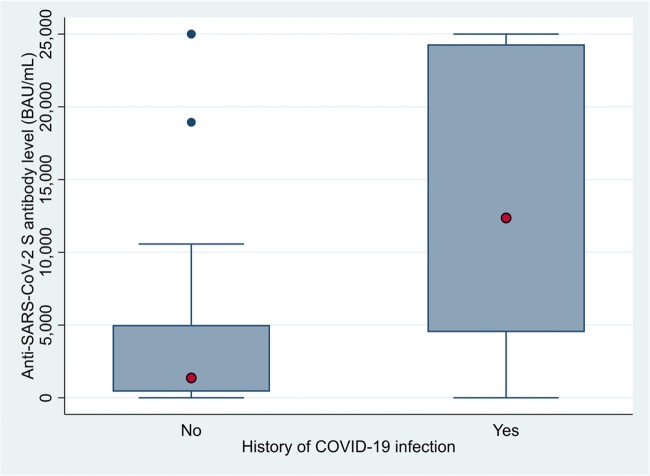

**Conclusion:**

This study showed a high anti-SARS-CoV-2 S antibody response in KTR after the 4^th^ vaccine dose, with 73% achieving a good response at > 6 months. Prior COVID-19 infection was associated with a good antibody response, indicating role of hybrid immunity among this patient population.

**Disclosures:**

**All Authors**: No reported disclosures

